# Home Cancer Care Research: A Bibliometric and Visualization Analysis (1990–2021)

**DOI:** 10.3390/ijerph192013116

**Published:** 2022-10-12

**Authors:** Boutheina Fhoula, Majed Hadid, Adel Elomri, Laoucine Kerbache, Anas Hamad, Mohammed Hamad J. Al Thani, Raed M. Al-Zoubi, Abdulla Al-Ansari, Omar M. Aboumarzouk, Abdelfatteh El Omri

**Affiliations:** 1 Division of Engineering Management and Decision Sciences, College of Science and Engineering, Hamad Bin Khalifa University, Doha 34110, Qatar; 2 Pharmacy Department, National Center for Cancer Care & Research, Hamad Medical Corporation, Doha 3050, Qatar; 3 Ministry of Public Health, Doha 31666, Qatar; 4 Surgical Research Section, Department of Surgery, Hamad Medical Corporation, Doha 3050, Qatar; 5 Department of Biomedical Sciences, College of Health Sciences, QU-Health, Qatar University, Doha 2713, Qatar; 6 Department of Chemistry, Jordan University of Science and Technology, P.O. Box 3030, Irbid 22110, Jordan; 7 College of Medicine, QU-Health, Qatar University, Doha 2713, Qatar; 8 School of Medicine, Dentistry and Nursing, The University of Glasgow, Glasgow G12 8QQ, UK

**Keywords:** cancer, home healthcare, home care services, bibliometric analysis, science mapping, knowledge domain, knowledge base, social structure, intellectual structure, conceptual structure

## Abstract

Home cancer care research (HCCR) has accelerated, as considerable attention has been placed on reducing cancer-related health costs and enhancing cancer patients’ quality of life. Understanding the current status of HCCR can help guide future research and support informed decision-making about new home cancer care (HCC) programs. However, most current studies mainly detail the research status of certain components, while failing to explore the knowledge domain of this research field as a whole, thereby limiting the overall understanding of home cancer care. We carried out bibliometric and visualization analyses of Scopus-indexed papers related to home cancer care published between 1990–2021, and used VOSviewer scientometric software to investigate the status and provide a structural overview of the knowledge domain of HCCR (social, intellectual, and conceptual structures). Our findings demonstrate that over the last three decades, the research on home cancer care has been increasing, with a constantly expanding stream of new papers built on a solid knowledge base and applied to a wide range of research themes.

## 1. Introduction

Cancer is a major public health concern all over the world and is the leading cause of early mortality and disability [1], with significant psychological, social, and economic implications for patients and societies [2,3,4]. In 2018, cancer costs totaled USD 199 billion in Europe. Total costs included health care costs, informal care costs, drugs, and productivity losses from early mortality and morbidity [5]. In 2015, cancer also accounted for a considerable share of total healthcare spending in the United States, as USD 165 billion and USD 18 billion was spent on cancer-related health care and drugs, respectively, totaling USD 183 billion, with that figure expected to rise by 34%, to reach USD 246 billion by 2030 [6]. Cancer costs will undoubtedly rise in the future, given the rising incidence and prevalence of the disease, along with enhanced detection and treatment options.

Furthermore, great strides have been made in the domains of surgical and radiologic oncology, resulting in therapies with reduced complications and burden on patients [7]. However, systemic therapies (targeted and immunotherapy) lag behind in this regard, and cancer care facilities are therefore jeopardized by the high number of hospital visits resulting from current systemic therapies. In addition, more and more patients are requiring more treatment approaches, chronic care, and longer follow-up. These therapies are also emotionally distressing, and patients’ social functioning is harmed because of their high frequency and chronic nature [2]. Additionally, over the last few decades, there has been significant growth in understanding cancer’s psychological effects, and the need for preserving the health-related quality of life while undergoing anti-cancer therapies and afterward [8]. Thus, more resources are needed to provide ongoing patient education and enhanced psychosocial care.

To address the aforementioned cancer care issues, the healthcare system is shifting away from its historically dominant focus on the inpatient setting, and developing more adaptable, cost-effective, and patient-centered models of cancer care. Home healthcare (or “home care”) is one important application of such new models [9]. Even though home healthcare is becoming more popular, the concept behind this care system might be vague, and executed in various ways in different countries [10]. The World Health Organization defines home healthcare, as a system of care provided at the patient’s home to help avoid or delay the need for acute or long-term institutional interventions [11]. 

Most medical needs for people of all ages are met in the comfort of their own homes within a home healthcare system. This system of care provides a variety of services, from the provision of medical supplies to more complex medical interventions such as nursing, nutritional support, physiotherapy, occupational and speech therapy, and pain management [12,13].

In general, home healthcare is regarded as an effective health system for optimizing healthcare costs by lowering the number and length of hospital stays, and a means of providing better-integrated care [14]. Furthermore, remaining in a familiar environment for a long time is thought to enhance patient quality of life [14].

Over the last decade, research focusing on home cancer care has steadily increased. So far, the related research has been summarized in several literature review studies focusing on the type of home-based intervention, such as end-of-life care for terminal cancer patients [15], nutritional interventions [16,17], exercise program or physical activity interventions [18], and chemotherapy [19,20]. Other review papers have focused on home intervention outcomes, such as health outcomes [21], patient experience [22,23], caregiver experience [15,24], safety [25], and cost [7]; meanwhile, others have focused on specific cancer types, such as lung cancer [26] and hematologic malignancies [27], or on specific populations such as pediatric cancer patients [28,29] or caregivers [30]. 

To the best of our knowledge, although many research studies have highlighted key elements of HCCR, no comprehensive knowledge mapping of the whole research area has been attempted; that is, there have been no studies that explore the social, intellectual, and conceptual structures of this field.

As the home cancer care (HCC) field is continuously expanding in size and scope, it is difficult to provide a global view of this field’s research patterns and topics through manual and intellectual examination. Herein, we use the bibliometric analysis to conduct a review of the HCCR. This technique is a quantitative and comprehensive method that applies bibliometrics to analyze large scholarly data sets, map the structure and progress of research fields, and predict the evolution of various disciplines [31,32]. Furthermore, bibliometric analysis tries to evaluate the intellectual landscape of a knowledge area and identify problems that academicians have been striving to answer, as well as the methods they have developed to achieve their goals, through network modeling and visualization [33]. 

The goals of this study were to explore the general trends in HCCR, to outline the major contributions to the field considering countries, institutions, journals, and authors, to establish the relationships or social structure, to identify the foundational literature, and to investigate the primary research themes. The study findings will help researchers build knowledge and gain the understanding needed to further their investigations.

## 2. Materials and Methods

### 2.1. Data Retrieval

Data were retrieved from Scopus, Elsevier’s abstract and citation database. Scopus is the largest academic database, with sophisticated tools for tracking, analyzing, and visualizing research from more than 23,452 peer-reviewed journals and 210,000 books, as well as 9.8 million conference papers and over 77.8 million records [34]. In addition, it is the most comprehensive database on the HCCR topic, providing all the data needed for quantitative research [35]. 

Defining a search strategy is essential in order to find a comprehensive set of papers on a specific research topic, improve the accuracy of the search and simplify large data gathering [36,37]. In the current study, a collection of search keywords was used to extract literature from the Scopus database, with an emphasis on (a) the care setting (home), (b) the disease or population (cancer patient), and (c) the intervention or type of care. The search strategy is listed in Table S1.

### 2.2. Data Screening

**Figure 1:**
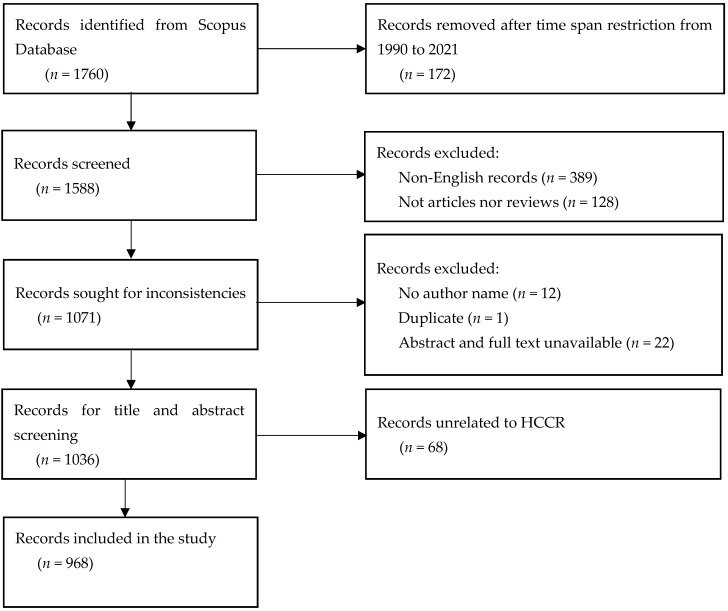
Flowchart of the data screening process.

The search strategy, without establishing a time restriction, yielded 1760 publications (as of April 2021). Although HCCR has a long history reaching back to 1948, in this study we were interested in the most recent advancements in this vibrant research field, particularly during the past three decades (1990–2021). In addition, to prevent errors caused by variations in data presentation formats, only English language articles and reviews were included in the sample; duplicates and papers with missing information were excluded. Furthermore, two researchers carefully screened the title and abstract to verify that they were linked to HCCR, and in case of a disagreement, a third researcher was consulted. A total of 968 out of 1760 research papers satisfied all of the criteria. Figure 1 shows the data screening flow chart of this study. 

### 2.3. Data Preprocessing

Before carrying out the data analysis, we had to preprocess the data collected from the Scopus database by supplementing missing content and eliminating inconsistencies for different attributes such as authors, affiliation data (country and institutions), author keywords, and cited references.

For authors appearing under different names (e.g., “Benthien K.”, “Benthien K.S.”; “Addington-Hall J.”, “Addington-Hall J.M.”; and “Chu S.H.”, “Chu S.-H.”), a disambiguation process was performed, and several author names were merged. For country data, we replaced cities with their corresponding countries, added missing ones, unified country names (e.g., “UK”, “United Kingdom”; and “USA”, “United States”), and eliminated inconsistencies. In addition, different affiliations to the same high-level organization are considered distinct by most bibliographic software; this inconsistency in affiliation data can be explained by lower hierarchical levels appearing in the first part of affiliations (e.g., departments and units), the same affiliation expressed in different languages (e.g., “bologna university”, “università di bologna”), and the use of abbreviations (e.g., “ices, Canada”, “institute for clinical evaluative sciences, Canada”). Therefore, for preprocessing affiliation data, we replaced abbreviations, removed data noise such as addresses, reduced the organizational level of each affiliation to its highest level such as university, hospital, research center, association, or firms, and finally clustered and reconciled the different identified main organizations. For author keywords, we fixed misspellings (e.g., “terminal illnes”), eliminated hyphens and apostrophes (e.g., “end of life care”, “end-of life care”), merged American and British spellings (e.g., “program evaluation”, “programme evaluation”), changed Latin spellings (e.g., “leukaemia”, “leukemia”), standardized near-identical words (e.g., “hospital based home care”, “hospital care at home, ” “deaths at home”, “die at home”), changed plural forms to singular, and merged synonyms to commonly used terms (e.g., “surgery”, “surgical procedure”, “surgical resection”). Some compound keywords were divided into parts (e.g., “dementia and cancer” was divided into “dementia” and “cancer;” “supportive and palliative care” was divided into “supportive care” and “palliative care;” “transfusion of elderly hematological patient” was divided into “transfusion”, “elderly”, and “hematological patient”).

Additionally, in network analysis, nodes of degree one (referred to as pendant nodes) of low-frequency keywords are typically considered to be of lower value and are occasionally filtered away. Thus, to avoid losing all the information concerning specific and low-frequency keywords, we linked them to a broader and higher frequency group, to preserve some of the keywords’ information. As an example, “cost-effectiveness” was divided into “cost” and “cost-effectiveness;” similarly “cost minimization” was divided into “cost” and “cost minimization”. 

Finally, cited references included journal publications, technical reports, handbooks, software, web links, manuals, policies, procedures, and guidelines, among others. However, the same cited reference may be supplied in a variety of formats, and it may contain ambiguities and inconsistencies of various kinds, such as in the number and citation style of cited authors, source title, source volume and issue, and page number. In general, most bibliometric software is unable to recognize these inconsistencies and fails to cluster the different formats of the same cited reference, and consequently, their citations are not added. To preprocess cited references, we removed noise from each reference (such as PubMed ID, DOI, access date, links) to reduce the cited reference to its main constituent elements (author names, article title, publication year, source title, source volume and issue, and page number). Subsequently, a clustering process was performed using combinations of match keys (name of the first author, publication year, and begin page number). This necessitated a thorough, time-consuming, and meticulous review of all the cited references, and it was a highly challenging task to clean and cluster more than 27,801 items.

### 2.4. Data Analysis and Visualization

Bibliometrics were used to analyze and represent the data, such as the number of articles and citations, the ratio of citations per article, year of publication, Hirsch index (h-index), Journal Impact Factor (JIF) from JCR 2020, and Total Link Strength. 

**Figure 2:**
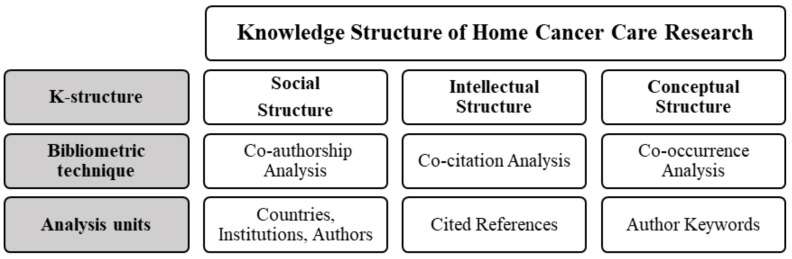
Knowledge Structure Analysis of Home Cancer Care Research.

Analysis of the knowledge structures (k-structures) using several bibliometric techniques was performed to investigate the social structure or collaboration networks among authors, institutions, and countries, explore the intellectual structure of the knowledge base, and depict the conceptual structure or major research topics. The analysis of the k-structures of HCCR was carried out using VOSviewer (version 1.6.16), as explained in Figure 2. VOSviewer is a visualization software used to visualize bibliometric networks and scientific maps [38]. 

## 3. Results

### 3.1. Publication Trends

**Figure 3:**
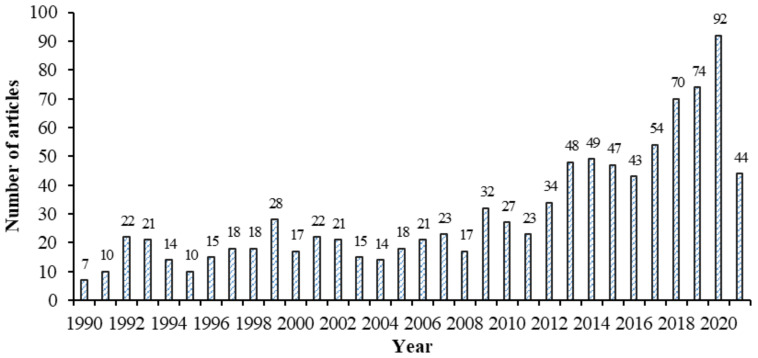
Annual publication trend (1990–2021).

In general, the number of articles on HCC increased throughout the last three decades, from 7 articles in 1990 to 92 articles in 2020, with an average of 29.8 articles per year. For the year 2021, 44 articles on HCC were published as of April 2021 (Figure 3). During the 19 years from 1990 to 2008 particularly, there was slow growth, with only 331 relevant published articles, with an average of 17.42 articles per year. Subsequently, the annual number of articles grew substantially to 32 in 2009 and jumped significantly to 92 articles in 2020. This growth trend indicates that the development of HCC is drawing the attention of a growing number of researchers, practitioners, academicians, and other players.

### 3.2. Analysis of Countries

**Figure 4:**
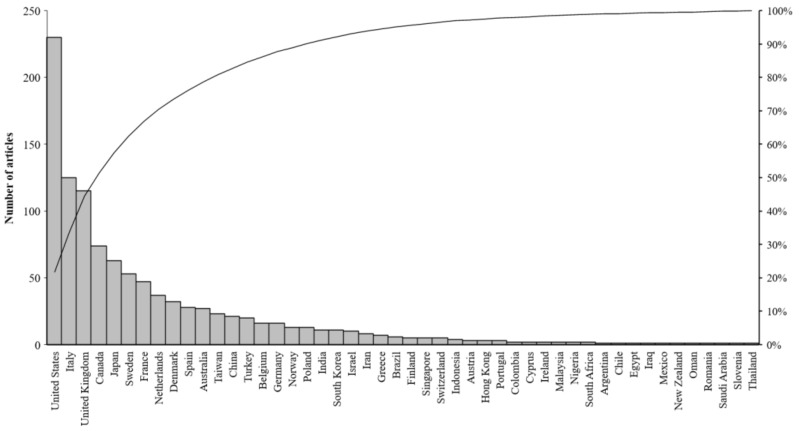
Scientific production by country.

In general, the 968 articles on HCC were published by 48 countries. Figure 4 shows the scientific contribution of these countries.

By far the most publications were generated in the United States (230/968, 23.76%). Two countries, Italy (125/968, 12.91%) and the United Kingdom (115/968, 11.9%), produced between 101 and 200 articles. A total of 17 countries (34.69%) produced between 11 and 100 articles, and 29 countries (59.18%) produced 10 articles or fewer.

Table 1 lists the top ten most productive countries, which published 76.45% of the total articles (740/968). Among these countries, seven are European, two are from North America, and one is from East Asia. The United States was the most productive country (n = 230), accounting for 23.76% of the total, followed by Italy, the United Kingdom, Canada, Japan, Sweden, France, the Netherlands, Denmark, and Spain.

Similarly, documents from the United States had the most citations (n = 6161) and the highest h-index. This demonstrates that the United States is not only the most active country in HCCR but also the most influential in terms of published literature. However, Sweden, which ranked sixth in number of publications and fourth in h-index and number of citations, had the highest average citations per publication.

**Figure 5:**
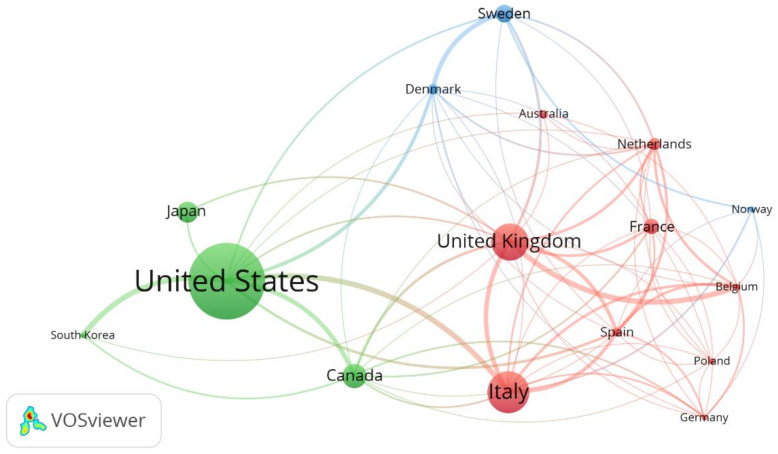
Countries’ co-authorship network map. Threshold of a minimum number of 5 articles and a minimum Total Link Strength of 5. The normalization method was Linlog/modularity. The weight was the number of documents per country. The line thickness indicates the strength of the collaboration relationship. Same color of nodes indicates that they belong to the same countries’ cluster.

VOSviewer was used to map the countries’ co-authorship network as displayed in Figure 5. Only 16 countries out of the 48 met the defined thresholds and were grouped into three clusters. The first cluster includes the United States, Canada, Japan, and South Korea. The second cluster includes Italy, the United Kingdom, France, the Netherlands, Germany, Belgium, Poland, and Australia, while the third cluster includes Sweden, Denmark, and Norway. 

### 3.3. Analysis of Institutions

A total of 1482 institutions from the 48 countries were actively involved in HCCR, with a high level of collaboration. Of these institutions, 78.9% (n = 1170) originate from only 12 countries (United States, United Kingdom, Italy, France, Japan, Canada, Netherlands, Spain, Sweden, Australia, Taiwan, and Germany). Table 2 lists the ten most productive institutions publishing on HCC from 1990 to 2021. 

Almost all these institutions were universities, and they (co-)published 14.56% of all articles in this research field (140/968). The University of Toronto leads the ranking of the ten most productive research institutions, followed by Karolinska Institute and La Maddalena Cancer Center. These leading institutions serve as key publishing hubs for HCCR throughout the world. 

**Figure 6:**
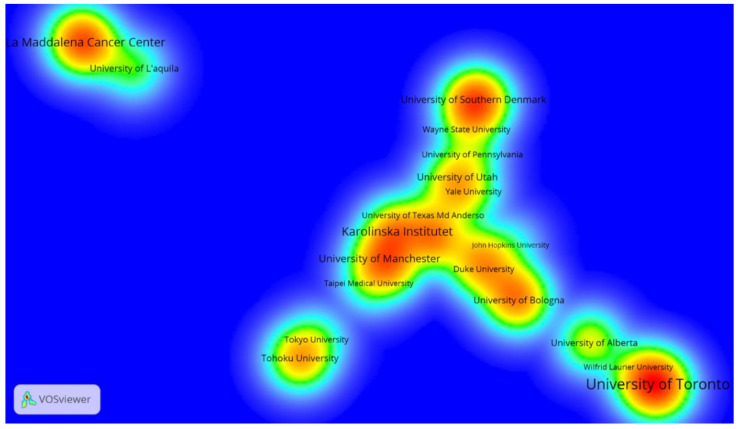
Institutions’ co-authorship density map. Threshold of a minimum number of five papers. The normalization method was Linlog/modularity. The weight was the Total Link Strength. The color of a node ranges from blue to green to yellow to red and is determined based on the density of items at that node. The higher the node density, the closer its color to red. In reverse, the lower the node density, the closer its color to blue. The number of items in the neighborhood as well as their weights determines item density.

Relationships of collaboration between research institutions were also considered, and VOSviewer was used to generate the institutions’ co-authorship density visualization map (Figure 6).

Only 81 institutions out of 1482 met the defined threshold, and most of them have developed several stable collaboration networks. In addition, identification of the nodes with high density shows that institutions such as the University of Toronto, La Maddalena Cancer Center, and the University of Southern Denmark, have performed major roles in the research network and have contributed significantly to the advancement of HCCR.

### 3.4. Analysis of Authors

The 968 articles were authored by 4024 researchers. More than 80% of authors (3306/4024) published only one paper; 429 published two papers (10.66%); 275 published between three and nine (6.83%) and 14 published ten or more (0.35%). Table 3 displays the 10 most productive authors publishing articles related to HCC. 

Italian researchers stand out, with six of the top ten most productive authors and 99 articles, followed by Swedish (two authors, 23 articles), Danish (one author, 12 articles), and British (one author, 10 articles) researchers. The top three authors by number of publications and h-index are Mercadante S., Aielli F., and Casuccio A., and they are all affiliated with Italian institutions. Mercadante S. published the largest number of articles, most of them as the first author and had an average of 32.9 citations per paper and a Total Link Strength of 125. However, Higginson I.J., ranked ninth in number of articles and sixth in h-index, and was the most cited author as well as the one with the highest average citations per publication.

**Figure 7:**
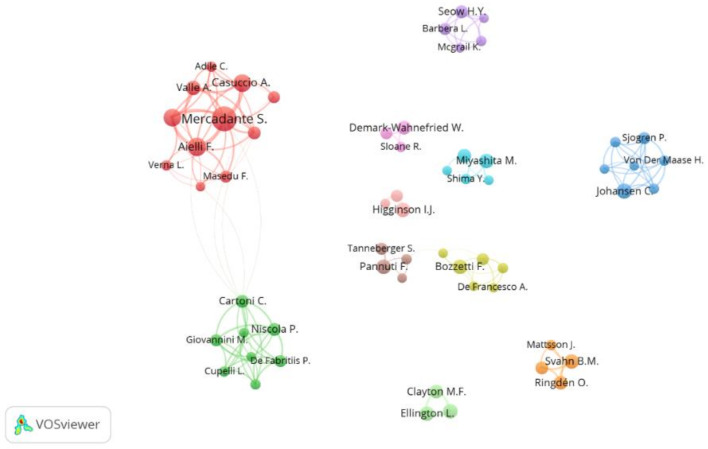
Author’s co-authorship network map. Threshold of a minimum number of five articles and a minimum Total Link Strength of five. The normalization method was Linlog/modularity. The weight was citations. The line thickness indicates the strength of cooperation relationship. Same color of nodes indicates that they belong to the same authors’ cluster.

VOSviewer was used to map the authors’ co-authorship network, as visualized in Figure 7. This resulted in a network of 59 nodes or authors, divided into 11 clusters of collaborative researchers, 146 co-authorship links, and a Total Link Strength of 723. One can observe that these collaboration networks are stable, as many authors collaborated closely within a fixed network based on the same research interests. In addition, the top four authors in number of publications belong to one cluster of the collaboration network and collaborate more frequently.

### 3.5. Analysis of Journals

Journal analysis was useful for determining the most important journals on the subject of HCC. The selected 968 articles related to this subject were published in 349 journals over 30 years. Most journals published only one paper on HCC, and only a small number published ≥ 20 papers (Table 4).

Table 5 lists the top ten journals in terms of the number of papers published from 1990 to 2021; these journals published 30.27% (293/968) of all articles included in this study. The top ten journals were all published in three developed countries (Germany, United Kingdom, and United States). The average impact factor of these ten journals was 2.3049 (JCR 2020).

The Journal of Pain and Symptom Management, Supportive Care in Cancer, and Palliative Medicine were the only three journals that published ≥ 40 papers, suggesting that studies about HCC are viewed favorably in these three journals. The top five journals belong to the first quartile (Q1 JCR in 2020). In general, quartiles and JIF values for the top ten journals were high. The periodical with the greatest impact factor was Palliative Medicine, with 3.739 (JCR 2020). Psycho-Oncology, although ranked last in number of articles, had the second-highest number of citations per article.

### 3.6. Analysis of Articles and Citations

The citation of a paper measures its quality, visibility, and impact within a research field. It can also describe the academic field’s research hotspots. The literature on HCC has a structure in which a limited number of publications account for a large percentage of the citations. Out of the 968 articles, six had more than 200 citations each, 33 had between 100 and 199 citations (3.41%), 175 had between 25 and 99 citations (18.07%), and 372 had between 5 and 24 citations (38.42%). However, 39.46% of published articles had fewer than five citations, or were not even cited. Table S2 analyses the general citation structure of HCC literature.

Table 6 illustrates the 10 most cited articles. The average number of citations was 270.1, but only three articles were cited more than 270 times. The majority (n = 8) of these articles were published between 2000 and 2010. 

### 3.7. Analysis of Cited References

There were 20,489 cited references; the most frequently cited references were the ones by Higginson and Sen-Gupta [48], Gomes and Higginson [39], Aaronson et al. [49], Zigmond and Snaith [50], and Gomes et al. [51]. Table S3 lists the top 20 most cited references.

Co-citation analyses were also employed to analyze cited references. A co-citation link between two papers is established when they are cited jointly by a source paper. The greater the number of co-citations a pair of papers receives, the stronger their co-citation strength is, and the more semantically connected they are. 

**Figure 8:**
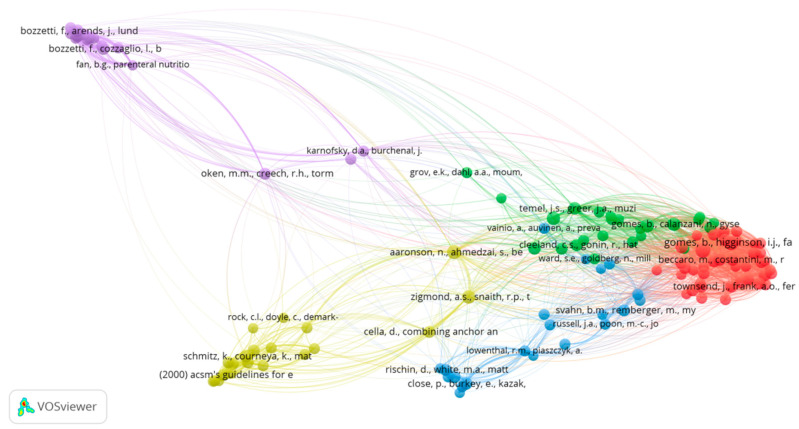
Cited references co-citation network map. Minimum number of citations set to 10. The normalization method was Linlog/modularity. The weight was citations of the reference. The line thickness indicates the strength of the co-citation link. Same color of nodes indicates that they belong to the same cluster.

The co-citation network of the references with a minimum number of 10 citations, resulted in a network of 126 nodes, 1835 co-citation links, and a Total Link Strength of 4489, as visualized in Figure 8. The co-citation map depicted five clusters, each representing a different research line or investigation theme of the knowledge base of HCCR.

### 3.8. Analysis of Keywords

This analysis aimed to determine the most common keywords used to categorize articles on HCC and explore their distribution, using co-occurrence analysis. Only authors’ keywords were considered in this analysis. The selected 968 documents related to HCC included 1062 author keywords, of which 727 appeared once, accounting for 68.46% of the total; 302 appeared between 2 and 19 times (28.44%); 28 appeared between 20 and 99 times (2.64%) and 5 appeared more than 100 times (0.47%). 

Apart from the main research terms (“cancer”, “home care”, and “home”), “palliative care” was the most recurrent keyword for HCC articles, followed by “quality of life” and “caregiver”. Table S4 lists all of the keywords that appeared more than 20 times (top 32 keywords).

**Figure 9:**
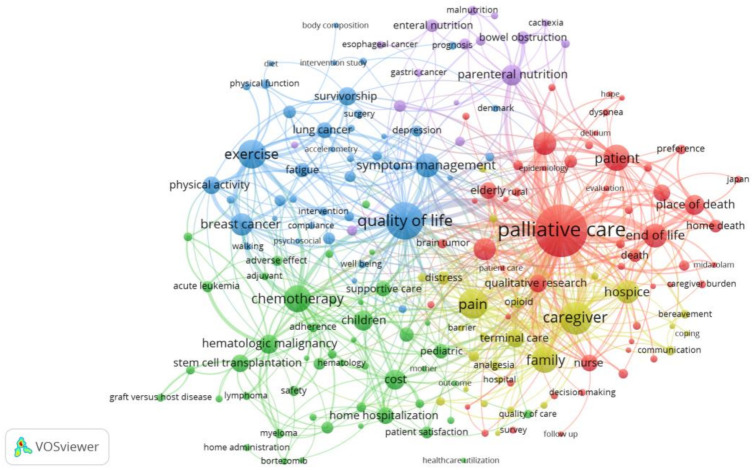
Keywords co-occurrence network map. Minimum occurrences of an author keyword were set to three. Generic terms “cancer”, “home care”, “home” and “care” were excluded. The normalization method was Linlog/modularity. The weight was the occurrences of keywords. The line thickness reflects the co-occurrence link strength. Same color of nodes indicates that they belong to the same cluster.

A keywords co-occurrence network map was extracted using VOSviewer software, resulting in a network of 183 nodes that met the defined thresholds, 1625 co-occurrence links, and a Total Link Strength of 3137, as visualized in Figure 9. The 183 keywords were separated into five groups or clusters, each represented by a color. These clusters reflect mainstream research issues and topics in the field of HCC. 

## 4. Discussion

In this study, we performed bibliometric and visualization analyses on HCC by searching publications in the Scopus database within a 31-year time period (1990–2021). Even though several studies have highlighted significant aspects of HCCR, no thorough mapping of the entire field has been performed. This paper is pioneering, in that it presents mapping and analyzing of the knowledge structure of the HCCR field.

### 4.1. Main Information

The quantitative analysis of the 968 retrieved papers yielded several results, which we will discuss below. In the first place, even though HCCR has been conducted regularly since the 1990s, we observed a recent successful period of academic literature linked to this topic, namely from 2009 to April 2021. More than 166 papers on the subject were published within the last two years of the investigation period. This upward trend suggests that HCC is attracting the attention of an increasing number of researchers, practitioners, and academicians, and this research field will likely continue to advance at a rapid pace. However, HCCR has not received equal attention around the world, although several countries have published papers on the subject. In this research field, the United States leads the way, with notable contributions from European countries such as the United Kingdom, Italy, and Sweden. These findings are consistent with the prevalence of home care provision systems or practices in developed countries. 

Furthermore, the analysis of main institutions shows that the University of Toronto, Karolinska Institute, and La Maddalena Cancer Center have played key roles in the research network and made substantial contributions to the advancement of HCCR. We observed that the research in this field is particularly active in Canada, with four institutions in the top ten; in addition, the University of Alberta, although ranked last based on total publications, is ranked first and fifth based on average citations and h-index, respectively, indicating that its contributions have a high impact and quality. In the same way, the University of Palermo, ranking fourth with 18 articles, has a total citation, average citation, and h-index that ranked third, second, and first, respectively, confirming that the published articles by Italian institutions are also highly influential in HCCR.

Mercadante S. is the leading author in terms of both publications and collaboration, with 30 published papers, 988 citations (32.9 citations per paper), and a Total Link Strength of 125. Mercadante S. is currently a unit director in La Maddalena Clinic for Cancer and a palliative medicine professor at the University of Palermo. In addition, he is recognized as a world-renowned expert in cancer palliative care. In 2013, he received the “John Mendelson MD Award” from MD Anderson Cancer Center [52]. Mercadante S. has published many papers on pain and symptoms management during supportive and palliative care [53,54,55]. On the other hand, Higginson I.J. from King’s College London, United Kingdom, was the most cited author, with 10 articles and 1027 citations (102.7 citations per paper). Higginson I.J.’s research topics of interest center on place of death and end-of-life care for terminal cancer patients [56,57]. 

HCC is a complicated topic that is indeed of interest in many disciplines, and therefore the number of journals that have covered the topic is significant. This large number of sources from many study disciplines provides an opportunity for authors to find a home for their publications. However, the most productive journals in this field were Journal of Pain and Symptom Management, Supportive Care in Cancer and Palliative Medicine, while Psycho-Oncology was the second most impactful journal in number of citations per article.

The journal Supportive Care in Cancer focuses on medical, technological, and surgical aspects related to cancer supportive care [58]. The Journal of Pain and Symptom Management publishes the most recent clinical findings and best practices linked to the reduction of burden in patients suffering from serious or life-threatening illnesses [59]. Palliative Medicine is committed to expanding palliative care research and clinical practice for terminally ill patients [60], while Psycho-Oncology is dedicated to all of cancer’s psychological, social, behavioral, and ethical implications [61]. 

One can observe that all these journals are medical ones, and their subject areas are medicine, nursing, and psychology. No journals from decision science or engineering figured in the main sources, suggesting that operation management and optimization problems are understudied in this research field.

The subject’s importance was also reflected in high number of citations (19,298 citations for 968 articles). However, we observed that the literature on HCC has a structure in which a small number of publications account for a substantial proportion of overall citations. Approximately 10% of all articles (100/968) received more than 53% of total citations, with more than 50 citations per article. In addition, around 40% of published articles had fewer than five citations or were not cited at all. This might indicate one of two things: either they are recent articles, or they have a low academic impact and are not relevant enough to be considered. 

Citation analysis revealed that the three most prominent and referenced publications in HCC ranked in the top three in total citations, as well as in citations per year. The paper by Gomes and Higginson [39], which ranked first, studied the impact of several variables on the place of death in oncologic patients. The second most cited paper was the one by Morey et al. [40] which examined whether combined dietary and exercise interventions can help older and overweight cancer survivors reorient their functional degradation. The paper by Pinto et al. [41], which ranked third, investigated the effectiveness of an at-home physical activity intervention in promoting psychological well-being and supporting recovery in early-stage breast cancer patients. 

### 4.2. Social Structure of HCC Research

Cooperation or interchange of disciplines in an area is a crucial aspect of scientific research, and it arises from the problem’s complexity, knowledge growth dynamics, and subject knowledge professionalism [62]. Cooperation might be reflected by a thorough examination of the interactions of various countries, institutions, and authors. 

This study shows that most countries cooperated, and the more regular exchanges a country developed, the higher was its production. The main clusters in the co-authorship map are led by four countries (United States, Italy, United Kingdom, and Sweden). Furthermore, the United States collaborated more with countries from North America (Canada) and East Asia (South Korea and Japan), whereas Italy and the United Kingdom collaborated more with countries from Western Europe, such as France, Netherlands, Germany, and Belgium (second cluster). Other countries from Northern Europe have also played a significant role, especially Sweden, Denmark, and Norway (third cluster).

Furthermore, in addition to studying the academics’ productivity, visualizing their collaborative networks is also important. A single author can rarely supply all the knowledge and resources required to handle major or fundamental scientific concerns. In addition, teams tend to publish more influential research than individuals [63]. The co-authorship analysis illustrates 11 clusters or collaboration networks of researchers who collaborated closely, based generally on the same research topic. The analysis also reveals the development of several stable collaboration networks between institutions.

### 4.3. Intellectual Structure of the HCC Knowledge Base

Scientific knowledge accumulates or builds up over time, which means that new knowledge is developed based on previously acquired knowledge [64]. As a result, the word “knowledge base” or “intellectual base” refers to the concepts, viewpoints, techniques, theories, and methodologies employed in the generation of new knowledge in a certain scientific topic. References to prior literature are commonly employed in the scientific literature as a proxy for the knowledge utilized, and therefore for the knowledge base. In bibliometric terms, references to prior literature are the “cited articles” and constitute the knowledge base, while the “citing articles” serve as the research front.

Through network analysis, the co-citation analysis splits cited articles into clusters, allowing visualization and investigation of the research field’s structure, characteristics, relationships, and evolution [65]. As co-citation analysis is a bibliometric method that assumes papers that are frequently referenced together are thematically related, clusters of co-cited papers are thematic clusters [66]. In that way, co-citation analysis can disclose the primary intellectual structure of a research domain and provide useful information for scientific edge-cutting spots [67]. However, co-citation analysis focuses solely on highly cited articles and excludes recent or niche ones from its theme clusters. In this way, co-citation analysis is appropriate for researchers seeking to investigate major themes or research lines, in addition to locating the most important publications or “foundational literature” [68]. 

In this study, the underlying intellectual structure of the HCC knowledge base was explored and disclosed through co-citation analysis of highly cited references. Five clusters were represented on the network co-citation map, each reflecting a different research line or investigation theme of this research field. For each of these five clusters, we assigned a suitable marker based on a review of the titles of all individual publications and studied the two key or foundational documents. 

#### 4.3.1. Place of Care for Terminal Cancer Patients

Cluster 1 (red) represents publications mainly dealing with the end-of-life place of care. Various related research topics were discussed, such as patient preference, family perspective, and impact on the place of death. For the home care alternative, papers discussed the impact on patient and family caregivers’ quality of life. Some other publications dealt with the place of death (hospital vs. home) and studied the related patient preference, family perspective, challenges, factors and predictors, and degree of agreement between the actual and preferred place of death.

The first key document in this cluster is the paper by Higginson and Sen-Gupta [48], with 53 citations and the highest Total Link Strength of 2092, thus occupying a key position in the knowledge base. The authors in this paper systematically appraised the evidence of the cancer patients’ and caregivers’ preferences concerning the location of terminal care and death and the differences between these preferences and those of the wider public. In the case of advanced disease, home care was the most popular option, followed by hospice care. The study highlighted the fact that meeting these needs could have a big impact on how services are delivered.

The second key document in this cluster is the one by Gomes and Higginson [39], with 53 citations and Total Link Strength of 1913. The authors in this paper investigated the effect of several factors on the location of death in oncologic patients. The network of these factors was found to be convoluted. The paper suggested that future legislation and clinical practice should prioritize measures to enhance home care, help families and the public to gain knowledge, skills, and resources, as well as educate and train practitioners in end-of-life care.

#### 4.3.2. Exercise or Physical Activity Intervention for Cancer Patients

Cluster 2 (yellow) is mainly about exercise or physical activity intervention during cancer therapies, especially for breast cancer patients. The related publications studied the exercise’s impact on fatigue, physical functioning, quality of life, and emotional distress. Other publications presented scales to assess cancer symptoms (hospital anxiety and depression scales, and anemia and fatigue scales), or presented various exercise guidelines for cancer survivors. 

The first key document in this cluster is the paper by Aaronson et al. [49], with 35 citations and Total Link Strength of 1435. In this paper, Aaronson et al. presented the outcomes of an international field study using the EORTC QLQ-C30 questionnaire designed in 1986 to evaluate the quality of life of patients taking part in clinical trials.

The second key document in this cluster is the one by Zigmond and Snaith [50], with 32 citations and Total Link Strength of 1399. In this paper, the authors proposed the HADS (Hospital Anxiety and Depression Scale), a self-assessment scale for non-psychiatric patients to assess psychological discomfort. This scale has shown excellent psychometric qualities in a variety of groups, such as cancer inpatients [69] and nursing home residents without cognitive impairment [70]. Zigmond and Snaith [50] stated that incorporating the HADS into regular hospital practice would make easier the difficult process of detecting and managing emotional disorders in patients undergoing medical and surgical examinations and treatments.

#### 4.3.3. Home Palliative Care for Advanced Cancer

The green Cluster 3 mainly relates to home palliative care for advanced cancer patients. The related papers can be separated into two sub-clusters; the first one concerns the effectiveness, cost-effectiveness, assessment systems, and effects on clinical outcomes of home palliative care. Meanwhile, the second one follows the family and informal caregivers of advanced cancer patients at home; it studies their preferences and perspectives, their needs and concerns, as well as their experiences.

The first key document in this cluster is by Gomes et al. [51], with 28 citations and Total Link Strength of 1081. In this systematic review, Gomes et al. investigated how estimations of a preference for home death varied, and examined the causes for variation, mainly in terms of study quality, preference measurement method, and how these preferences changed as the illness progressed. The authors observed that although the majority would prefer to die at home, many of them still do not die at home. The paper underlined the necessity of increased actions on previously discovered factors influencing home death, for more people’s wishes to be fulfilled. The paper also recommended additional research in order to learn more about the factors that influence mortality at home.

The second key document in this cluster is by Gomes et al. [71], with 27 citations and Total Link Strength of 1126. In this paper, the authors determined first the impact of home palliative care on the likelihood of a home death for adult patients with advanced disease, and on various patient and caregiver outcomes (quality of life, symptom management, caregiver distress, and satisfaction). The paper also assessed the resource use and costs, and thoroughly evaluated and summarized the present cost-effectiveness evidence. The findings of the study provided solid evidence that this home intervention enhanced the likelihood of a home death and reduced symptom burden while not affecting caregiver distress. The findings supported providing palliative care at home for people who prefer a home death.

#### 4.3.4. Home Parenteral Nutrition for Advanced Cancer

Cluster 4 (purple) mainly relates to home parenteral nutrition (HPN) for advanced cancer patients, especially for those with malignant gastrointestinal obstruction. Various related themes were discussed, such as nutrition guidelines, nutritional outcomes, impact on quality of life, prognosis and survivorship, patient experience, and family caregiver experience.

The first key document in this cluster is the paper by Bozzetti et al. [72], with 26 citations and Total Link Strength of 885. In patients whose regular food consumption is insufficient and enteral feeding is impractical, considered unsafe, or not approved by the patient, parenteral nutrition has the potential to augment or assure nutrient consumption. However, for terminal cancer patients, the administration of HPN has sparked heated controversy in Europe. HPN is a costly and time-consuming intervention, and there is little evidence that it extends survival in these patients. It is also unknown whether HPN can improve quality of life, regardless of overall survival. Through an observational prospective study, Bozzetti et al. [72] investigated changes in quality of life in oncologic patients receiving HPN, as well as the potential relationship between clinical and oncological markers, and length of survival. The paper recommended the use of HPN for chronically obstructed and malnourished cancer patients, who failed to respond to traditional therapies, as well as for patients with a Karnofsky performance > 50. 

The second key document in this cluster is the one by Bozzetti et al. [73], with 22 citations and Total Link Strength of 689. In this paper, the authors presented evidence-based guidelines on the use of parenteral feeding for oncologic patients. The guidelines were created using the most relevant papers from the previous three decades, and they echoed ESPEN guidelines on enteral nutrition in oncological patients.

#### 4.3.5. Home Chemotherapy

Finally, publications in Cluster 5 (blue) focused mainly on home chemotherapy, especially for pediatric oncology patients. The related publications evaluated many aspects, such as preference, satisfaction, outcomes, and quality of life of patients, as well as economic impact, cost analysis, compliance, and safety. Other publications evaluated the home care provided after stem cell transplantation (during the pancytopenia phase). This cluster is led by three key documents. The first one is by Close et al. [74], with 20 citations and Total Link Strength of 463. Administration of chemotherapy at home is a viable alternative to hospitalization for pediatric cancer patients; however, only a few home chemotherapy programs for children have been reported or reviewed in the literature. In this paper, Close et al. compared in-hospital chemotherapy to at-home chemotherapy in terms of charges billed, out-of-pocket expenses, wage loss, clinical outcomes, and quality of life. 

The second key document in this cluster is by Svahn et al. [75], 17 citations and Total Link Strength of 672. In this paper, the authors provided evidence that home care of stem cell transplantation patients during the pancytopenia phase was an innovative and risk-free intervention. According to this paper, home care offered various benefits, including reduced need for parenteral feeding, earlier discharge, lower cost, and lower transplantation-related mortality.

The third key document in this cluster is by Borras et al. [76], with 17 citations and Total Link Strength of 540. In this paper, the authors compared at-home versus outpatient delivery of chemotherapy for colorectal cancer patients in terms of healthcare resource utilization, quality of life, safety, and adherence. Home chemotherapy appeared to be a viable and safe alternative to institutional settings with the potential to improve treatment adherence and satisfaction.

### 4.4. Conceptual Structure of HCC Research

Keywords in research papers convey information about their core content [77]. An examination of the main keywords and relational analysis is useful to investigate the knowledge or conceptual structure of a research field, as well as to investigate current and potential research hotspots [78]. While document co-citation analysis helps explore the intellectual structure of the knowledge base (foundational literature), co-occurrence analysis is used to investigate the conceptual structure or research topics in a research field and their interactions with different fields [37,79]. In addition, co-occurrence analysis is the only bibliometric technique that constructs a measure of similarity based on the content of the documents through keywords, whereas the other methods link papers indirectly through citations or co-authorship [79].

In this study, the conceptual structure of the HCC knowledge domain was explored and disclosed through author keywords co-occurrence analysis, with a threshold of three occurrences. The selected 183 keywords were separated into five groups or clusters. These clusters reflected mainstream research concepts and topics in the HCCR field.

#### 4.4.1. Home Palliative Care 

The first of these clusters, or Cluster 1 (red), comprises 53 keywords, and the main ones include “palliative care”, “end-of-life”, “patient”, “advanced cancer”, “nursing care” and “place of death”. We observed that the largest node was “palliative care”, which appeared in over a quarter of the papers in our database, and it is the node with the largest Total Link Strength. Thus, palliative care is the focus of the most comprehensive and in-depth research studies on HCC. Palliative care is the comfort care provided to advanced cancer patients who require a high level of supportive care. A growing body of evidence suggests that home palliative care could reduce symptom burden, increase understanding of illness and prognosis, and improve patients’ quality of life and overall survival [80,81]. On the other hand, many other studies focused on the impact of palliative care location on the end-of-life experience for terminally ill patients and their family caregivers, especially death experience [82,83].

#### 4.4.2. Home Care and Chemotherapy

Cluster 2 (green) comprises 46 keywords and the main ones include “chemotherapy”, “children”, “cost”, “hematologic malignancy”, “stem cell transplantation”, and “supportive care”. 

Chemotherapy for cancer patients was traditionally administered in a hospital setting. However, there has recently been a transition in chemotherapy treatment from inpatient to outpatient ambulatory therapy and home therapy. A growing number of studies are evaluating home chemotherapy as an alternative to hospitalization, especially for pediatric cancer patients. Studies focused on different outcomes, such as cost [84,85], adherence [86], safety [87], compliance, satisfaction, and quality of life [76].

#### 4.4.3. Home-Based Exercise Intervention

The main keywords of Cluster 3 (blue, 41 items) include “quality of life”, “exercise”, “symptom management”, “breast cancer”, “survivorship”, and “physical activity”. Many cancer survivors endure lasting physical, mental, and emotional problems because of their diagnosis and treatment, affecting their quality of life [2,88]. Physical activity is suggested as a potential strategy for coping with cancer diagnosis and therapy side effects, and intervention for the rehabilitation of cancer patients in particular during palliative care [89]. According to a growing body of studies, significant gains in physiologic and psychosocial functioning have been shown in adult cancer survivors who participate in exercise programs [90]. Despite the established benefits, several legal, organizational, and patient-related challenges impede the implementation of exercise programs in cancer survivors, such as lack of specialized rehabilitation programs, lack of referral from clinicians, and low awareness on the part of patients [91,92]. 

Home exercising can alleviate such accessibility constraints that prevent cancer survivors from taking part in standard institutional programs under professional supervision. A growing number of studies provide evidence that home exercise intervention is feasible and safe [93,94], and is subsequently a promising alternative to institutional interventions.

#### 4.4.4. Caregiver Experience

The main keywords of Cluster 4 (yellow, 26 items) include “caregiver”, “family”, “hospice”, “pain”, “terminal care”, and “distress”. Home care for cancer patients is mostly dependent on a family caregiver, as has been well established in the literature [95]. As professional caregivers seldom visit the home, and for short periods, the primary responsibility of home care for an advanced cancer patient lies generally with the family. One of the most challenging duties for many family caregivers is pain management [96,97], which can cause caregivers to become distressed and dissatisfied, lowering their quality of life, as they experience difficulties carrying out these tasks [98]. Thus, effective pain management will reduce patient suffering while also easing the burden on family caregivers [99]. 

#### 4.4.5. Home-Based Nutritional Support

The main keywords of Cluster 5 (purple, 17 items) include “parenteral nutrition”, “bowel obstruction”, “enteral nutrition”, “nutrition”, and “nutrition support”. Malnutrition is a common medical problem among oncological patients that harms survivorship and quality of life [100,101]. Thus, nutritional support is generally recommended as adjunctive therapy for cancer patients [102] and varies from diet counseling to enteral or parenteral nutrition [103]. Various observational studies have demonstrated that home-based nutritional support is beneficial for patients after anti-cancer therapy and that at-home artificial feeding (either enteral or parenteral feeding) is strongly advised for patients with persistent nutritional deficiency [104,105].

Despite the established benefits, the prevalence of home nutritional intervention differs around the world, due to differences in healthcare policies, organizational and legislative structure, as well as to many other economic, social, and ethical factors that impede the use of home nutritional interventions for cancer patients [106,107].

### 4.5. Future Research Directions

Based on the analyses provided in this paper and the previous discussion, there are several potential future research areas that have not yet been addressed. Firstly, the obtained results from the conceptual clustering have revealed that the current trends of HCCR tend to focus on separate home care interventions or services (palliative, end of life, chemotherapy, nutritional support and exercise), with the main emphasis on assessing the impact of the different programs. However, the potential synergistic effects of these interventions have not been thoroughly studied. 

Secondly, for each service, most studies try to expose the subject and present evidence on the care adherence, the different humanistic outcomes (such as HRQoL, satisfaction and preference) and the economic outcomes (cost and resource utilization) using descriptive (surveys, qualitative) and analytic (experimental and observational) studies. However, less attention has been paid to the organizational aspect and the operational management of HCC. The literature on home health care operations management is well developed, whereas few papers have tackled the specificity of cancer care, focusing mainly on care coordination between home operations and conventional hospital care operations. In contrast to other medical conditions, home-based cancer care necessitates a multidisciplinary approach from a variety of skilled professionals, including oncologists, pharmacists, caregivers, nutritionists and psychologists, as well as an integrated approach to solve the logistical problems at different decisions levels (strategic, tactical and operational). Home cancer care is nowadays facing operational difficulties all over the world, and research in operations management can offer then significant solutions to these problems, using simulation techniques and optimization models. 

Thirdly, due to the aging population, health care systems all over the world will need to deal with a rapidly rising number of cancer patients requiring home palliative care, but few of these systems seem ready to meet the special needs of this population. Digital support and telecommunication technology allows these challenges to be addressed, and patients can then receive more care at home, through mobile communications, teleconferencing, and electronic patient-reported outcome (ePRO) monitoring systems. However, despite the advancement and use of technology in home care research in general, authors have shown less interest in this area for cancer. The need to study the integration of technology into home cancer care is also motivated by the current shift of cancer care models toward a more patient-centered approach, where patients’ perspectives, preferences and needs are prioritized, in particular their preferences for telehealth care or remote home care. 

Therefore, local authorities should support and steer such initiatives by establishing laws and financing schemes that support these holistic and patient-centered healthcare models, and a thorough analysis of the difficulties and potential enablers associated with implementing telehealth care would be crucial for future home cancer care planning. Furthermore, there is a need for studies that focus on multimodal HCC and study the synergistic impact. Moreover, more research needs to integrate the use of operational management approaches and techniques to address the current issues that HCC is facing.

## 5. Limitations and Future Research Directions

There are few potential limitations to mention despite the contribution of this study. Firstly, the authors relied mostly on Scopus as a database to extract the literature. This choice is mainly motivated by the fact that Scopus is the largest abstract and citation database of peer-reviewed literature. Using multiple databases may lead to a more comprehensive literature, particularly if we want to extend the scope of the paper beyond engineering management to capture more social, behavioral, or clinical aspects of Home Cancer Care. 

The second limitation is mainly related to the type of publications that were evaluated. In the HCC literature, we omitted conference papers, book chapters, notes, and letters. Although this search strategy gave us the most important and relevant works, additional studies could build on this one by examining other types of secondary papers in the Scopus database in order to spot other pioneering tendencies or expand the analysis to other sources and data sets that evaluate different types of reports, such as Ph.D. theses. Furthermore, we only analyzed papers written in English, which undervalues research conducted in other languages. Papers written in Japanese (the second most-used language in the number of published papers related to HCC) were not included, suggesting that key HCC studies in Asia may have been omitted. In addition, the search results are limited, since the search keywords were only used in the “Title ( )” Scopus search option. Using the “Title-abstract-keywords ( )” search option yielded over 11,000 results, many of which were unrelated to our study subject. HCC is a cross-cutting topic, and we did not want to detract from the study’s focus by including works that were not directly relevant to the field.

Moreover, one can enrich this study by using additional bibliographic or mapping analysis allowed by the choice of software such as bibliographic coupling and co-citations for cited authors and journals, or using other software or methods that provide different analyses such as time-slicing and burst-detection analysis. The research might also be expanded by refining the study with a more in-depth examination of the identified clusters and topics. In addition, future studies are required to map future changes in the conceptual structure and primary research topics of this research area over time. 

## 6. Conclusions

The home cancer care field is continuously expanding in size and scope, and many research studies highlighted key elements of HCCR. Conversely, no comprehensive knowledge mapping of the whole research area has been attempted.

In this paper, we conducted a bibliometric and visualization analysis of the HCCR field, to investigate the status and the structure of its knowledge domain (social, intellectual, and conceptual structures). We first explored the general trends in terms of published papers and outlined the major contributions, considering countries, institutions, journals, and authors. Following this, we established the collaboration or social structure among these entities, using co-authorship analysis. We also identified the foundational literature, using co-citation analysis and investigated the primary research topics, using co-occurrence analysis. 

Our findings demonstrate that over the last three decades, the research on home cancer care has steadily increased, with a constantly expanding stream of new papers built on a solid knowledge base and applied to a wide range of research themes. The knowledge base (foundational literature) of HCCR has concentrated on five primary areas of concern: the place of care for terminal cancer patients, exercise or physical activity intervention, home palliative care and home parenteral nutrition during end-of-life care, and home chemotherapy. The five major clusters of research topics were home palliative care, home care and chemotherapy, home-based exercise intervention, caregiver experience, and home-based nutritional support.

The findings of this study will help academicians in this research area gain a better comprehension of the current state of knowledge needed to further their research, and help policymakers and practitioners support informed decision-making about new HCC programs.

## Figures and Tables

**Table 1:** Top Ten Most Productive Countries Publishing on Home Cancer Care (1990–2021).

Rank	Countries	Region	Publications (P)	%	Citations (C)	C/P	h-Index
1	United States	North America	230	23.8	6161	26.8	43
2	Italy	Southern Europe	125	12.9	3110	24.9	30
3	United Kingdom	Northern Europe	111	11.9	3141	27.3	29
4	Canada	North America	74	7.6	1466	19.8	21
5	Japan	East Asia	63	6.5	542	8.6	13
6	Sweden	Northern Europe	53	5.5	1526	28.8	21
7	France	Western Europe	47	4.9	664	14.1	14
8	Netherlands	Western Europe	37	3.8	588	15.9	14
9	Denmark	Northern Europe	32	3.3	396	12.4	12
10	Spain	Southern Europe	28	2.9	411	14.7	9

Data are presented as numbers or percentages.

**Table 2:** Top Ten Most Productive Institutions Publishing on Home Cancer Care (1990–2021).

Rank	Institution	Country	Publications (P)	%	Citations (C)	C/P	h-Index
1	University of Toronto	Canada	34	3.51	347	10.2	12
2	Karolinska Institute	Sweden	23	2.38	693	30.1	14
3	La Maddalena Cancer Center	Italy	22	2.27	705	32.0	15
4	University of Palermo	Italy	18	1.86	685	38.1	15
5	University of Manchester	United Kingdom	16	1.65	475	29.7	9
6	McMaster university	Canada	15	1.55	224	14.9	10
7	University of Southern Denmark	Denmark	15	1.55	176	11.7	7
8	Institute for Clinical Evaluative Sciences	Canada	14	1.45	89	6.4	6
9	University of Utah	United States	14	1.45	268	19.1	7
10	University of Alberta	Canada	13	1.34	635	48.8	11

Data are presented as numbers or percentages.

**Table 3:** Top Ten Most Productive Authors Publishing on Home Cancer Care (1990–2021).

Rank	Author	Country	Institution	Publications (P)	%	Citations (C)	C/P	h-Index	TLS
1	Mercadante S.	Italy	La Maddalena Cancer Center	30	3.10	988	32.9	17	125
2	Aielli F.	Italy	University of L’Aquila	17	1.76	284	16.7	10	103
3	Casuccio A.	Italy	University of Palermo	16	1.65	614	38.4	13	73
4	Porzio G.	Italy	University of L’Aquila	15	1.55	280	18.7	10	90
5	Strang P.	Sweden	Karolinska Institute	12	1.24	339	28.3	9	26
6	Johansen C.	Denmark	Danish Cancer Society	12	1.24	118	9.8	7	87
7	Svahn B.M.	Sweden	Karolinska Institute	11	1.14	265	24.1	7	67
8	Pannuti F.	Italy	National Tumor Association Foundation	11	1.14	82	7.5	5	38
9	Higginson I.J.	United Kingdom	King’s College London	10	1.03	1027	102.7	8	62
10	Bozzetti F.	Italy	University of Milan	10	1.03	451	45.1	7	67

Data are presented as numbers or percentages. TLS: co-authorship Total Link Strength.

**Table 4:** Productivity of Journals.

Number of Articles per Journal	Number of Journals	%
1	227	65.04
2	47	13.47
3 or 4	38	10.89
Between 5 and 9	17	4.87
Between 10 and 19	12	3.44
Between 20 and 39	5	1.43
≥40	3	0.86
Total	349	100

Data are presented as numbers or percentages.

**Table 5:** Top Ten Most Productive Journals Publishing on Home Cancer Care (1990–2021).

Rank	Source	Publisher	Country	Publications (P)	%	Citations (C)	C/P	JIF	JIF Qu.
1	Supportive Care in Cancer	Springer Nature	Germany	68	7.0	1371	20.2	2.635	Q1
2	Journal of Pain and Symptom Management	Elsevier	USA	41	4.2	1341	32.7	3.077	Q1
3	Palliative Medicine	SAGE	UK	40	4.1	1931	48.3	3.739	Q1
4	Cancer Nursing	Wolters Kluwer Health	USA	25	2.6	811	32.4	1.85	Q1
5	European Journal of Cancer Care	Wiley-Blackwell	UK	22	2.3	230	10.5	2.161	Q1
6	BMC Palliative Care	Springer Nature	UK	22	2.3	146	6.6	2.015	Q2
7	Journal of Palliative Care	SAGE	USA	21	2.2	470	22.4	1.2	Q3
8	American Journal of Hospice and Palliative Medicine	SAGE	USA	16	1.7	80	5.0	1.638	Q3
9	Oncology Nursing Forum	Oncology Nursing Society	USA	16	1.7	618	38.6	1.728	Q2
10	Psycho-Oncology	Wiley-Blackwell	UK	14	1.4	644	46.0	3.006	Q1

JIF: Journal Impact Factor from the 2020 Journal Citation Reports (JCR 2020); JIF Qu.: JIF Quartile; data are presented as numbers or percentages.

**Table 6:** Top Ten Most Cited Articles Related to Home Cancer Care.

Rank	Article	Publication Year (Y)	Citations (C)	C/Y
1	Gomes and Higginson [39]	2006	650	43.33
2	Morey et al. [40]	2009	343	28.58
3	Pinto et al. 2005 [41]	2005	302	18.88
4	McCorkle et al. [42]	2000	244	11.62
5	Courneya et al. [43]	2003	219	12.17
6	Benzein et al. [44]	2001	206	10.30
7	Talcott et al. [45]	1994	199	7.37
8	Hinton [46]	1994	196	7.26
9	Bee et al. [15]	2009	185	15.42
10	Cohen et al. [47]	2010	157	14.27

Data are presented as numbers or percentages.

## Data Availability

All relevant data are within the manuscript and its Supporting Information Files.

## Supplementary Materials

The following supporting information can be downloaded at: https://www.mdpi.com/article/10.3390/ijerph192013116/s1, Table S1. Search Strategy for Home Cancer Care; Table S2. General Citation Structure of Home Cancer Care Literature; Table S3. Top Twenty Most Cited References; Table S4. Top Keywords with Occurrence > 20. References [108,109,110,111,112,113,114,115,116,117] are cited in the Supplementary Materials.
