# Supplementary material for: Home Cancer Care Research: A Bibliometric and Visualization Analysis (1990–2021)
Source: Int J Environ Res Public Health. 2022 Oct 12;19(20):13116. doi: 10.3390/ijerph192013116 (PMC9603182; doi:10.3390/ijerph192013116)
Supplement: Supplementary file 1 [file ijerph-19-13116-s001.zip › ijerph-1883441-supplementary.pdf]

International Journal of
Environmental Research and Public Health

MDPI

Table S1. Search Strategy for Home Cancer Care.

Scopus (April 3, 2021)		
**Number**	**Concept**	**Search strategy**
#1	Setting	home OR homecare
#2	Disease/Population	cancer OR anticancer OR oncology OR oncologic* OR oncologycal OR tumor OR chemotherapy OR hematology OR haematology OR hematologic* OR haematologic* OR (oncologic* W/0 "and" W/0 hematologic*) OR (oncologic* W/0 "and" W/0 haematologic*) OR (oncologic* W/0 hematologic*) OR (oncologic* W/0 haematologic*) OR hematooncology OR haematooncology OR hematooncologic* OR haematooncologic* OR hemato-oncology OR haemato-oncology OR hemato-oncologic* OR haemato-oncologic* OR oncohematology OR oncohaematology OR oncohematologic* OR oncohaematologic* OR onco-hematology OR onco-haematology OR onco-hematologic* OR onco-haematologic* OR lymphoma OR myeloma OR leukemia OR (blood PRE/0 cancer) OR (cell PRE/0 transplant*) OR (bone PRE/0 marrow PRE/0 transplant*) OR (malignant AND neoplasm)
#3	Intervention/Type of care	homecare OR care* OR caring OR healthcare OR intervention OR chemotherapy OR palliative OR pain OR treat* OR therapy OR dying OR death OR die OR infusion OR rehabilitation OR visit OR drug OR exercise OR administration OR medication OR injection OR transfusion OR monitoring OR *enteral OR symptom OR hospital* OR (physical AND activity) OR education OR ((followed OR treated OR managed) PRE/0 at PRE/0 home) OR (home PRE/0 management) OR pancytopenia OR pancytopenic OR neutropenia OR neutropenic OR prophylaxis OR prophylactic
Search strategy		TITLE (#1 AND #2 AND #3)

Pre/*n* and W/*n* are proximity operators to find words within a certain distance from each other; Pre/n specifies a word order whereas W/n does not.

International Journal of
Environmental Research and Public Health

Table S2. General Citation Structure of Home Cancer Care Literature.

Number of citations per article	Number of articles	%
≥ 600	1	0.10
≥ 300	2	0.21
≥ 200	3	0.31
≥ 100	33	3.41
≥ 50	61	6.30
≥ 25	114	11.78
≥ 5	372	38.43
< 5	221	39.46
Total	968	100%

Data are presented as numbers or percentages.

Table S3. Top Twenty Most Cited References.

Rank	Reference	Year	A	TLS
1	Higginson and Sen-Gupta [48]	2000	53	2092
2	Gomes and Higginson [39]	2006	53	1913
3	Aaronson et al. [49]	1993	35	1437
4	Zigmond and Snaith [50]	1983	32	1399
5	Gomes et al. [51]	2013	28	1081
6	Gomes et al. [71]	2013	27	1126
7	Bozzetti et al. [72]	2002	26	885
8	Beccaro [108]	2006	25	955
9	Temel et al. [109]	2010	25	815
10	Townsend et al. [110]	1990	23	704
11	Brumley et al. [111]	2007	22	915
12	Bruera et al. [112]	1991	22	796
13	Bozzetti et al. [73]	2009	22	689
14	Schmitz et al. [113]	2010	20	886
15	Close et al. [74]	1995	20	463
16	Grande et al. [114]	1998	19	802
17	De Conno et al. [82]	1996	19	590
18	Jordhoy et al. [115]	2000	19	825
19	Oken et al. [116]	1982	18	766
20	Ahlner-elmqvist et al. [117]	2004	17	737

A: Number of articles; TLS: Co-citation Total Link Strength, Data are presented as numbers or percentages.

International Journal of
Environmental Research and Public Health

Table S4. Top Keywords with Occurrence > 20.

Rank	Keyword	A	%	TLS	Rank	Keyword	A	%	TLS
1	Cancer	340	35.12	1660	17	Nursing care	40	4.13	200
2	Home care	242	25.00	1072	18	Care	39	4.03	209
3	Palliative care	217	22.42	1060	19	Parenteral nutrition	33	3.41	165
4	Home	147	15.19	756	20	Children	32	3.31	148
5	Quality of life	108	11.16	498	21	Cost	31	3.20	173
6	Caregiver	82	8.47	453	22	Place of death	31	3.20	140
7	Pain	68	7.02	342	23	Hematologic malignancy	29	3.00	136
8	Exercise	64	6.61	295	24	Survivorship	27	2.79	128
9	Chemotherapy	59	6.10	301	25	Terminal care	27	2.79	120
10	Family	56	5.79	315	26	Physical activity	25	2.58	106
11	Patient	54	5.58	255	27	Stem cell transplantation	25	2.58	95
12	End of life	45	4.65	216	28	Qualitative research	24	2.48	139
13	Symptom management	44	4.55	237	29	Elderly	23	2.38	116
14	Hospice	43	4.44	215	30	Nurse	23	2.38	122
15	Advanced cancer	42	4.34	192	31	Home hospitalization	22	2.27	110
16	Breast cancer	40	4.13	170	32	Terminal cancer	22	2.27	103

A: Number of articles; TLS: co-occurrence Total Link Strength, Data are presented as numbers or percentages.
